# Mechanism of Long-Term Corrosion Protection for Silicone Epoxy Coatings Reinforced by BN-PDA-CeO_2_ Ternary Composites in Harsh Environments

**DOI:** 10.3390/nano16020121

**Published:** 2026-01-16

**Authors:** Xianlian Mu, Tao Jin, Pengfei Xie, Rongcao Yu, Bin Li, Xin Yuan

**Affiliations:** 1 School of Aeronautics, Northwestern Polytechnical University, Xi’an 710072, China; 2 Key Laboratory of Corrosion Protection and Control of Aviation Technology, Special Vehicle Reach Institute, Aviation Industry Corporation of China, Ltd., Jingmen 448035, China; 3 School of Chemical Engineering and Technology, Sun Yat-Sen University, Zhuhai 519082, China

**Keywords:** BN-PDA-CeO_2_, silicone epoxy coating, harsh environment, long-term corrosion protection, multi-scale synergistic mechanism

## Abstract

Corrosion in harsh environments causes global economic losses exceeding 3 trillion US dollars annually. Traditional silicone epoxy (SE) coatings are prone to failure due to insufficient physical barrier properties and lack of active protection. In this study, cerium dioxide (CeO_2_) was in situ grown on the surface of hexagonal boron nitride (h-BN) mediated by polydopamine (PDA) to prepare BN-PDA-CeO_2_ ternary nanocomposites, which were then incorporated into SE coatings to construct a multi-scale synergistic corrosion protection system. Fourier transform infrared spectroscopy (FT-IR), X-ray diffraction (XRD), and transmission electron microscopy (TEM) confirmed the successful preparation of the composites, where PDA inhibited the agglomeration of h-BN and CeO_2_ was uniformly loaded. Electrochemical tests showed that the corrosion inhibition efficiency of the extract of this composite for 2024 aluminum alloy reached 99.96%. After immersing the composite coating in 3.5 wt% NaCl solution for 120 days, the coating resistance (Rc) and charge transfer resistance (Rct) reached 8.5 × 10^9^ Ω·cm^2^ and 1.2 × 10^10^ Ω·cm^2^, respectively, which were much higher than those of pure SE coatings and coatings filled with single/binary fillers. Density functional theory (DFT) calculations revealed the synergistic mechanisms: PDA enhanced interfacial dispersion (adsorption energy of −0.58 eV), CeO_2_ captured Cl^−^ (adsorption energy of −4.22 eV), and Ce^3+^ formed a passive film. This study provides key technical and theoretical support for the design of long-term corrosion protection coatings in harsh environments such as marine and petrochemical industries.

## 1. Introduction

Metal corrosion is a common and costly problem in harsh environments, causing global economic losses of over 3 trillion US dollars annually [1]. Although organic coatings are widely used as the main corrosion protection measure due to their cost-effectiveness and ease of application, they have two core limitations: first, insufficient physical barrier—micropores and microcracks formed during curing provide fast diffusion channels for corrosive media such as water, oxygen, and chloride ions (Cl^−^), leading to coating delamination after 1–2 years of service [2,3,4]; second, lack of active repair capability—once mechanical damage occurs, the metal substrate is directly exposed and severe corrosion occurs rapidly within 3 months [5,6].

To address these issues, two-dimensional (2D) nanomaterials such as hexagonal boron nitride (h-BN) have attracted significant attention due to their unique “labyrinth effect” [7,8]. This effect enhances the barrier property of coatings by extending the diffusion path of corrosive media. Unlike other 2D materials such as graphene oxide (GO), h-BN has a wide band gap of approximately 6 electron volts (eV), exhibiting excellent electrical insulation [9,10,11]. This fundamentally avoids the galvanic corrosion problem that may be caused by GO, making it a research hotspot in the field of corrosion protection [1,7,12,13,14,15]. However, h-BN nanosheets are prone to agglomeration in polymer matrices due to strong van der Waals forces between layers, which seriously affects their dispersibility and performance in coatings [1,8,13]. To overcome this challenge, polydopamine (PDA), a bionic material, was introduced. Inspired by the adhesion ability of mussels, PDA can self-polymerize to form films on the surface of various materials. Surface modification of h-BN with PDA can effectively improve the dispersibility of h-BN in resin matrices [3,8,12,16,17] and enhance the interfacial compatibility between h-BN and polymer matrices, thereby improving the uniformity and integrity of coatings. For example, PDA-modified h-BN (PDA-BN) has been proven to effectively improve the corrosion protection performance of epoxy coatings. Although PDA can improve the dispersibility of h-BN, its main contribution is to enhance the physical barrier property of coatings, providing only passive protection. When macroscopic damage (such as scratches) occurs on the coating, PDA modification alone cannot provide active repair or corrosion inhibition functions [5,8,18,19].

To make up for the deficiency of passive protection, the introduction of rare earth oxide CeO_2_ is a key strategy. As an intelligent corrosion inhibitor, CeO_2_ has both chloride ion capture and active corrosion inhibition functions [4,20,21,22]. Studies have shown that Ce^3+^ ions in CeO_2_ can react with water and oxygen in corrosive environments to form a dense Ce(OH)_3_/CeO_2_ passive film on the metal surface, thereby effectively inhibiting further corrosion of the metal. At the same time, the unique oxygen vacancy structure of CeO_2_ enables it to effectively adsorb chloride ions, reducing the erosion of these corrosive ions on the metal substrate. For example, CeO_2_ nanoparticles have been successfully applied in PMMA-CeO_2_ coatings to achieve effective self-healing and corrosion protection performance [20]. In carbon nanotube-polydopamine-cerium dioxide (CeO_2_-PDA-CNTs) composite coatings, CeO_2_ significantly enhanced the corrosion barrier property of the coatings [5]. Compared with GO or MXene, the combination of CeO_2_ and h-BN avoids additional problems caused by poor material conductivity or air stability [1,6].

Constructing h-BN, PDA, and CeO_2_ into a ternary nanocomposite and applying it to silicone epoxy (SE) coatings can achieve multi-level and multi-mechanism synergistic corrosion protection effects. Currently, no studies have effectively combined these three materials into SE coatings; therefore, this research direction has important scientific value and engineering application potential. SE coatings themselves have excellent high-temperature resistance and weather resistance, making them ideal for applications in harsh environments [23]. The design of this ternary composite aims to significantly improve the long-term corrosion protection performance of SE coatings in harsh environments such as marine, petrochemical, and outdoor photovoltaic fields through the synergistic effect of physical barrier, interface regulation, and active corrosion inhibition. By optimizing the loading amount of h-BN, the modification degree of PDA, and the nanomorphology and dispersibility of CeO_2_, a new type of SE composite coating with excellent corrosion protection capability was developed.

The complementary nature of CeO_2_ and h-BN lies in their distinct and mutually reinforcing corrosion protection functions. h-BN, as a 2D layered material, constructs a physical barrier via the “labyrinth effect” to block the penetration of corrosive media (H_2_O, O_2_, Cl^−^), but it lacks active response capability to defects or corrosive ions. In contrast, CeO_2_ exhibits dual active functions: its oxygen vacancy structure efficiently captures Cl^−^ to suppress pitting corrosion, and Ce^3+^ released in corrosive environments forms a dense Ce(OH)_3_ passive film to repair local defects. This combination overcomes the limitations of single-component fillers—h-BN compensates for the poor long-term barrier property of CeO_2_, while CeO_2_ makes up for the lack of active protection of h-BN. Compared with binary composites (e.g., PDA-BN or CeO_2_-PDA), the ternary system integrates physical barrier, Cl^−^ capture, and active passivation, achieving “passive defense + active response” synergistic protection, which is crucial for long-term corrosion resistance in harsh environments.

In this study, CeO_2_ was in situ grown on the surface of h-BN mediated by PDA to prepare BN-PDA-CeO_2_ ternary nanocomposites, which were used as functional fillers to incorporate into SE coatings. This solves the problems of h-BN agglomeration and poor interfacial compatibility with SE resin; the “dispersion-bridging” dual functions of PDA realize the uniform dispersion of fillers. The SE coating is endowed with triple synergistic protection capabilities of “passive barrier-Cl^−^ capture-active corrosion inhibition”, breaking the limitation of “single protection” of traditional coatings. Combined with experimental characterization and DFT calculations, the molecular-level protection mechanism was revealed, providing theoretical support for the design of corrosion protection coatings in harsh environments.

## 2. Experimental Materials and Methods

### 2.1. Experimental Materials

Hexagonal boron nitride (h-BN, purity ≥ 99%) was purchased from Sinosteel New Materials Co., Ltd., Qingdao, China; dopamine hydrochloride (DA, purity ≥ 98%), hydrochloric acid (HCl, analytical grade), and absolute ethanol (C_2_H_5_OH, purity ≥ 99.5%) were all purchased from Shanghai Macklin Biochemical Co., Ltd., Shanghai, China; tris(hydroxymethyl)aminomethane (Tris, analytical grade) was purchased from Chengdu Ron Chemical Reagent Co., Ltd., Chengdu, China; cerium nitrate (Ce(NO_3_)_3_·6H_2_O, analytical grade) was purchased from Shanghai Aladdin Biochemical Technology Co., Ltd., Shanghai, China; hexamethylenetetramine (C_6_H_12_N_4_, analytical grade) was purchased from Nanjing Reagent Co., Ltd., Nanjing, China; deionized water (H_2_O) was self-made in the laboratory. Silicone epoxy (SE) resin refers to a copolymer of epoxy resin (E-51) and silicone resin (polydimethylsiloxane, PDMS) with a mass ratio of 3:1. AMEO is 3-aminopropyltriethoxysilane (CAS: 919-30-2), used as a curing agent for SE resin.

### 2.2. Preparation of BN-PDA-CeO_2_ Nanocomposites

#### 2.2.1. Preparation of PDA-Coated h-BN (BN-PDA)

**Figure 1:**
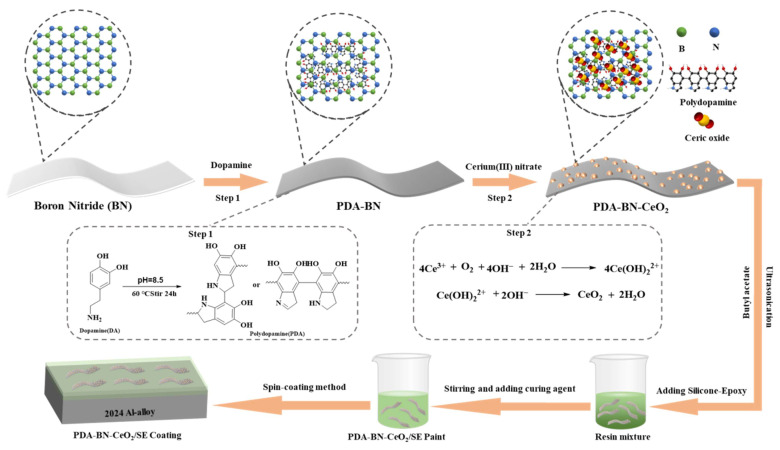
Schematic diagram of the preparation process of BN-PDA-CeO_2_ ternary nanocomposites and BN-PDA-CeO_2_/SE composite coatings (including the structural schematic of h-BN (B and N atoms are green and blue spheres, respectively), PDA-BN, and PDA-BN-CeO_2_ (CeO_2_ is yellow and red spherical clusters), the oxidative self-polymerization reaction path of dopamine (DA), the reaction formula of Ce^3+^ hydrolysis-oxidation to form CeO_2_, and the spin-coating preparation process of the composite coating).

0.5 g of hexagonal boron nitride (h-BN, 2D layered structure as shown in Figure 1, with B and N atoms represented by green and blue spheres, respectively) was weighed and added to 100 mL of Tris-HCl buffer solution (pH = 8.5, concentration 0.1 M, providing a weakly alkaline environment for DA polymerization); a 300 W probe sonicator (SCIENTZ-IID) was used for sonication for 30 min to fully exfoliate the h-BN layers and form a uniform suspension (ensuring layer dispersibility and providing a sufficient reaction surface for DA polymerization). 0.2 g of dopamine hydrochloride (DA) was added to the suspension, which was then placed in a constant temperature water bath at 30 °C (temperature fluctuation ± 0.5 °C) and magnetically stirred at 500 rpm for 24 h. During this process, DA undergoes oxidative self-polymerization under weakly alkaline conditions (pH = 8.5), and forms non-covalent interactions (π-π stacking, hydrogen bonding) or covalent bonding with the B-N bonds on the BN surface through the hydroxyl and amino groups of catechol, in situ forming a polydopamine (PDA) layer on the BN surface (as shown in the middle PDA-BN of Figure 1, where PDA molecules fill the interlayers of BN and modify the surface); at the same time, DA follows the reaction path shown in the figure: the catechol structure undergoes oxidation and cross-linking to gradually polymerize into PDA containing a large number of -OH and -NH_2_ functional groups, endowing the BN surface with hydrophilicity and reactivity. The reaction solution was centrifuged at 8000 rpm for 10 min using a Beckman Avanti J-E centrifuge (Beckman Coulter, Inc., Brea, CA, USA) to achieve solid–liquid separation; it was alternately washed 3 times with deionized water and absolute ethanol (purity 99.5%); finally, it was dried in an oven at 60 °C and a vacuum degree of 0.08 MPa for 12 h to obtain PDA-modified BN (BN-PDA) (after successful PDA coating, BN changes from hydrophobic to hydrophilic and remains well-dispersed after drying).

#### 2.2.2. Preparation of BN-PDA-CeO_2_ Composites

0.3 g of BN-PDA was weighed, dispersed in 50 mL of deionized water, and sonicated at 200 W for 15 min (to fully expose the -OH and -NH_2_ functional groups on the PDA surface and enhance the adsorption capacity for Ce^3+^); 20 mL of 0.1 M cerium nitrate solution (Ce(NO_3_)_3_·6H_2_O) was slowly added dropwise at a rate of 1 mL/min, and magnetic stirring was maintained at 300 rpm during the dropwise addition, with continuous stirring for 30 min. The -OH and -NH_2_ functional groups of PDA on the surface of BN-PDA undergo coordination with Ce^3+^ (as shown in the process of Ce^3+^ migrating to and adsorbing on the surface of PDA-BN in Figure 1), laying a foundation for the uniform growth of CeO_2_ in the subsequent step. The pH of the above mixture was adjusted to 9.0 using 0.1 M NaOH solution; the system was transferred to a 100 mL polytetrafluoroethylene-lined autoclave, placed in a program-controlled heating oven, and heated to 120 °C at a rate of 5 °C/min (slow heating to avoid uneven growth of CeO_2_ grains), with heat preservation for 6 h. During this process, Ce^3+^ undergoes chemical transformation following the reaction path shown in the figure: first, it hydrolyzes to form Ce(OH)_3_ (reaction formula: Ce^3+^ + 3OH^−^ → Ce(OH)_3_), and then Ce(OH)_3_ is further oxidized and crystallized to form CeO_2_ (reaction formula: 4Ce(OH)_3_ + O_2_ → 4CeO_2_ + 6H_2_O), finally forming uniformly distributed CeO_2_ nanoparticles on the surface of BN-PDA (as shown in the “PDA-BN-CeO_2_” on the right of Figure 1, where CeO_2_ is loaded on the surface of BN-PDA in the form of yellow and red spherical clusters). After the autoclave was naturally cooled to room temperature, the product was separated by centrifugation at 8000 rpm for 10 min; it was repeatedly washed with deionized water until the pH of the supernatant was 7 (to remove residual impurities such as Na^+^ and NO_3_^−^ and avoid affecting the subsequent coating performance); it was then dried in a vacuum oven at 60 °C for 12 h.

Hexamethylenetetramine (HMT) was not used for providing OH^−^ in this study. The pH of the mixture was solely adjusted to 9.0 using 0.1 M NaOH solution to trigger the hydrolysis and oxidation of Ce^3+^, ensuring the uniform growth of CeO_2_ nanoparticles. HMT was reserved as an alternative pH regulator but not applied in the final experimental process.

### 2.3. Preparation of BN-PDA-CeO_2_/SE Composite Coatings

2024 aluminum alloy (70 mm × 70 mm × 2 mm) was pretreated by grinding with 2000-mesh sandpaper → ethanol cleaning → drying; 0.1 g of nano-fillers (BN/PDA-BN/BN-PDA-CeO_2_) was dispersed in 1 g of butyl acetate (sonication for 30 min), and 10 g of SE resin and 2.5 g of curing agent AMEO were added (sonication for 15 min). The mass fraction of nano-fillers relative to the solid components (SE resin + AMEO) was calculated as follows: (0.1 g)/(10 g + 2.5 g) × 100% ≈ 0.8%. The spin-coating method was employed for coating preparation, with specific parameters: 800 r/min for 5 s to ensure uniform thickness. Air spraying was auxiliary used to repair local unevenness, followed by curing at 60 °C for 5 h and standing at room temperature for 7 days until complete curing, with a final coating thickness of 35 ± 5 μm. The control groups were pure SE coatings, denoted as SE, h-BN/SE, PDA-BN/SE, and BN-PDA-CeO_2_.

### 2.4. Characterization and Performance Test Methods

#### 2.4.1. Structure and Composition Characterization

A Fourier transform infrared spectrometer (FT-IR) (Thermo Fisher Nicolet iS50, Thermo Fisher Scientific Inc., Waltham, MA, USA) was used to collect spectra in the range of 4000–400 cm^−1^ to analyze the changes in functional groups. An X-ray diffractometer (XRD) (Bruker D8 ADVANCE, Bruker AXS GmbH, Karlsruhe, Germany) with Cu Kα radiation (λ = 1.5406 Å) as the light source was used, with a 2θ scanning range of 10–80°, to analyze the crystal structure and phase composition. A Raman spectrometer (Renishaw inVia, Renishaw plc, Wotton-under-Edge, Gloucestershire, UK, excitation wavelength 532 nm, laser power 5 mW, scanning range 50–3000 cm^−1^) was used to analyze molecular vibrations and structural defects. A thermogravimetric analyzer (TGA) (TA Instruments Q500, New Castle, DE, USA) was used to evaluate the thermal stability of nanocomposites and corresponding coatings under a nitrogen atmosphere (flow rate 50 mL/min) with a heating rate of 10 °C/min from 50 °C to 800 °C; the residual carbon rate at 800 °C reflects the high-temperature resistance of the material.

A scanning electron microscope (SEM) (Zeiss Sigma 300, Carl Zeiss AG, Oberkochen, Germany) was used to observe the macroscopic agglomeration morphology of the material, and combined with EDS spectroscopy and elemental mapping, the distribution uniformity of elements such as C, N, O, Ce, and B was analyzed. A transmission electron microscope (TEM) (FEI Talos F200X, Thermo Fisher Scientific (formerly FEI Company), Hillsboro, OR, USA) was used to observe the micro-fine structure of h-BN layers and CeO_2_ nanoparticles, and EDS was used to characterize the micro-distribution of elements such as P, C, N, O, and Ce, to visually verify the dispersibility of CeO_2_ in the BN-PDA matrix.

#### 2.4.2. Protection Performance Test

0.01 g of h-BN/PDA-BN/BN-PDA-CeO_2_ nano-fillers were dispersed in 200 mL of 3.5 wt% NaCl solution, stirred for 5 min → sonicated for 15 min → centrifuged at 3000 r/min for 5 min, and the supernatant (leachate containing uniformly suspended nanoparticles without obvious sedimentation) was collected to simulate the inhibition effect of active components and suspended particles released by nano-fillers on corrosion. Electrochemical tests adopted a three-electrode cell configuration: working electrode (WE) was 2024 aluminum alloy with an exposed area of 1 cm^2^ (sealed with epoxy resin except the test surface); reference electrode (RE) was Ag/AgCl (saturated KCl); counter electrode (CE) was a platinum sheet (1 cm × 1 cm). The test temperature was controlled at 25 ± 1 °C. Each sample was tested in 3 replicates to ensure data reliability. The equivalent circuit selection was based on the analysis of electrochemical behavior and fitting metrics (AIC, χ^2^).

A Corrtest CS350M electrochemical workstation (Wuhan Corrtest Instruments Corp., Ltd., Wuhan, Hubei, China) was used for electrochemical measurements. Prior to all tests, the working electrode was immersed in 3.5 wt% NaCl solution (temperature: 25 ± 1 °C) to stabilize the open-circuit potential (OCP) for 30 min. For electrochemical impedance spectroscopy (EIS) testing, measurements were performed at the stable OCP with a frequency range of 100 kHz–10^−2^ Hz, an AC amplitude of 20 mV, and testing after immersion for 60 h; equivalent circuit fitting was used to quantify the corrosion inhibition effect through oxide film resistance and charge transfer resistance. For polarization curve measurements, the potential range was set as −1.0 V to 0.2 V (vs. Ag/AgCl) with a scan rate of 1 mV/s, and Tafel extrapolation was used to obtain electrochemical parameters. Long-term corrosion test of intact coatings: immersed in 3.5 wt% NaCl solution for 120 days, and the barrier performance was evaluated by Nyquist plots and fitting parameters; XPS was used to characterize the corrosion products on the surface of aluminum alloy after immersion to verify the composition of the passive film.

#### 2.4.3. Verification of Molecular Mechanism (DFT Calculation)

The Vienna Ab-initio Simulation Package (VASP) 6.3.0 software was used to carry out density functional theory (DFT) calculations. The core purpose was to quantify the interfacial interaction energy in the passive barrier-Cl^−^ capture-active corrosion inhibition mechanism and reveal the molecular nature of ion transfer and adsorption. The calculation model was constructed to strictly match the experimental system: the h-BN model was a 4 × 4 × 1 supercell (containing 1 B vacancy, and the broadening and intensity reduction of the (002) peak in the XRD pattern of h-BN implied the presence of trace defects, which was further supported by TEM observations showing slight layer distortion) with a 15 Å vacuum layer on the surface; the PDA model selected dopamine monomers (self-polymerization units of PDA in the experiment), which were optimized and then adsorbed on the h-BN surface; the CeO_2_ model was a 3 × 3 × 1 CeO_2_(111) supercell (experimental HRTEM showed that CeO_2_ mainly exposes the (111) crystal plane), and 1 layer of Ce atoms was peeled off from the surface to form oxygen vacancies (experimental XPS confirmed the Ce^3+^/Ce^4+^ valence cycle). At the same time, adsorption systems such as PDA@h-BN, Ce^3+^@BN-PDA, Cl^−^@CeO_2_, and Ce^3+^-OH^−^@BN-PDA were constructed to simulate the core processes of the experiment. The calculation parameters were set as follows: the generalized gradient approximation (GGA) based on the Perdew-Burke-Ernzerhof (PBE) functional combined with DFT-D3 dispersion correction was used to describe the exchange-correlation interaction and van der Waals interaction; the projector augmented wave (PAW) pseudopotential was used to characterize the core-valence electron interaction; the cutoff energy was 500 eV; the Brillouin zone adopted a Γ-point centered 3 × 3 × 1 Monkhorst-Pack k-point grid; geometric optimization was carried out using the conjugate gradient algorithm, with termination conditions of single-atom force < 0.02 eV/Å and system energy convergence <1 × 10^−5^ eV. The analysis methods included calculating the adsorption energy using the formula $E_{\mathrm{ads}}=E_{\left(A/B\right)}-E_{A}-E_{B}$ (where $E_{\left(A/B\right)}$ is the total energy of the composite system, $E_{A}$ is the energy of the adsorbate, and $E_{B}$ is the energy of the substrate; a negative value indicates spontaneous adsorption), Bader charge analysis to determine the amount of interfacial charge transfer, and projected density of states (PDOS) to analyze orbital hybridization.

## 3. Results and Discussion

### 3.1. Characterization Results of BN-PDA-CeO_2_ Nanocomposites

#### 3.1.1. Chemical Bonding and Crystal Structure

To systematically clarify the chemical structure, phase, molecular vibration behavior, and thermal stability of hexagonal boron nitride (BN), PDA-modified BN (BN-PDA), and PDA-modified BN loaded with cerium dioxide (CeO_2_) (BN-PDA-CeO_2_), FT-IR, XRD, Raman spectroscopy, and TGA were used for characterization.

The FT-IR spectrum reflects the evolution of functional groups during material modification and CeO_2_ loading. For BN (red curve): the peak at 3427 cm^−1^ corresponds to the O-H stretching vibration of adsorbed water; the characteristic peaks at 1388 cm^−1^ and 892 cm^−1^ are attributed to the vibration of B-N bonds, confirming the intrinsic structural characteristics of hexagonal BN. For BN-PDA (green curve): a new peak appears at 1638 cm^−1^, corresponding to the stretching vibration of aromatic C=C bonds (derived from the aromatic rings in PDA), verifying the successful modification of PDA on the BN surface; the residual O-H peak also reflects the presence of hydroxyl/amino groups in PDA. For BN-PDA-CeO_2_ (blue curve): a significant peak appears at 565 cm^−1^, corresponding to the vibration of Ce-O bonds, directly proving the successful loading of CeO_2_ nanoparticles; at the same time, the characteristic peaks of BN and PDA are retained, indicating that the modification and loading processes do not damage the original structure.

The XRD pattern reveals the phase information of the material. For BN (red curve), the strong peak at 2θ ≈ 26.7° corresponds to the (002) crystal plane of hexagonal BN, confirming its typical hexagonal layered crystal structure. For BN-PDA (green curve): the (002) peak of BN still exists, but its intensity is slightly reduced—because PDA is an amorphous or weakly crystalline phase, which coats the BN surface and weakens the crystal diffraction signal, indirectly verifying the successful modification of PDA. For BN-PDA-CeO_2_ (blue curve): in addition to the (002) peak of BN, a series of diffraction peaks matching the cubic fluorite phase of CeO_2_ appear (e.g., 2θ ≈ 28.6° corresponds to the (111) crystal plane, 33.1° corresponds to the (200) crystal plane), indicating that CeO_2_ is successfully loaded on BN-PDA in a crystalline state.

The Raman spectrum reflects molecular vibrations and structural defects. For BN (red curve): the Raman signal is extremely weak because pure hexagonal BN has low Raman activity in this spectral range, and characteristic vibrations are difficult to excite. For BN-PDA (green curve): characteristic peaks of PDA appear at ~1364 cm^−1^ (D peak, induced by defects/disorder) and ~1579 cm^−1^ (G peak, stretching vibration of aromatic rings), confirming the successful modification of PDA on the BN surface. For BN-PDA-CeO_2_ (blue curve): in addition to the D and G peaks of PDA, a new peak at ~456 cm^−1^ corresponding to the vibration of Ce-O bonds is added, directly verifying the loading of CeO_2_; at the same time, the retention of PDA characteristic peaks indicates that the molecular structure of PDA is not damaged after CeO_2_ loading. BN’s E2g characteristic peak is located at ~1366 cm^−1^, which overlaps slightly with PDA’s D band (~1364 cm^−1^). To distinguish these features, Lorentzian function fitting was performed on the Raman spectrum of BN-PDA. The fitting results showed two separate peaks with a peak separation of ~2 cm^−1^, and the intensity ratio of the D band (PDA) to E2g band (BN) was ~0.8, confirming the presence of PDA on the BN surface. This result was cross-validated with FT-IR data (emergence of C=C stretching vibration at 1638 cm^−1^), consistent with literature reports of PDA modification on BN [12,13].

The TGA curve evaluates the thermal stability of the material. For BN (red curve): the mass loss is negligible in the entire temperature range, reflecting the excellent thermal stability of BN (almost no thermal decomposition). For BN-PDA (green curve): obvious mass loss occurs in the range of 200–450 °C, corresponding to the thermal decomposition of PDA (combustion/decomposition of organic components in PDA); this not only confirms the successful modification of PDA but also allows the estimation of its content through the mass loss ratio—the loading amount of PDA in BN-PDA is approximately 20%. For BN-PDA-CeO_2_ (blue curve): the mass loss is between that of BN and BN-PDA—because CeO_2_ is an inorganic oxide with good thermal stability, its loading inhibits the thermal decomposition of part of PDA, making the thermal stability of the composite better than that of BN-PDA. In summary, the series of characterizations fully verify the process from BN → BN-PDA (successful PDA modification) → BN-PDA-CeO_2_ (successful CeO_2_ loading with structure retention).

**Figure 2:**
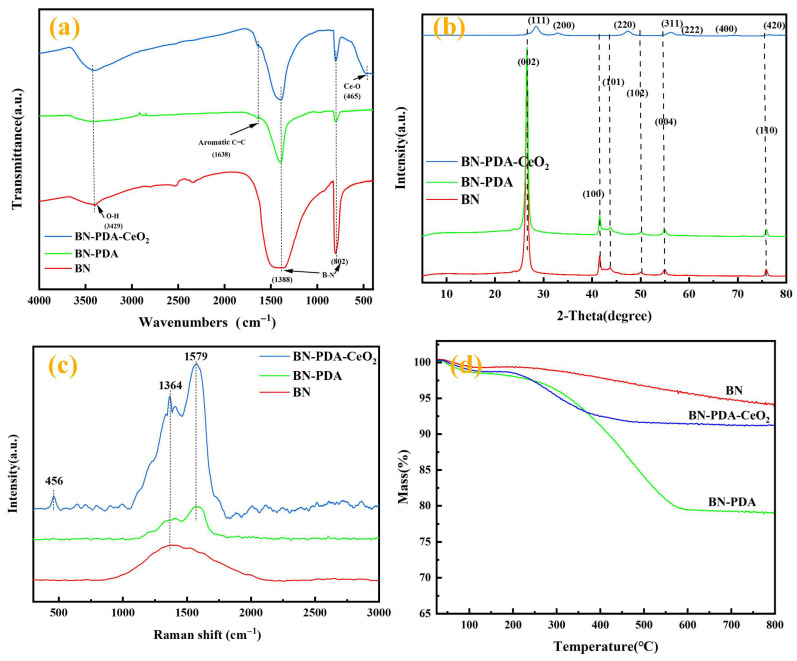
Structural characterization and thermal stability analysis of BN, BN-PDA, and BN-PDA-CeO_2_: (**a**) FT-IR spectra of BN, BN-PDA, and BN-PDA-CeO_2_, where the functional groups corresponding to each characteristic absorption peak (e.g., O-H, aromatic C=C, B-N, Ce-O, etc.) are labeled; (**b**) XRD patterns of BN, BN-PDA, and BN-PDA-CeO_2_; (**c**) Raman spectra of BN, BN-PDA, and BN-PDA-CeO_2_, showing the characteristic Raman shift peaks of each sample; (**d**) Thermogravimetric analysis (TGA) curves of BN, BN-PDA, and BN-PDA-CeO_2_.

This study’s FT-IR results showing the successful loading of CeO_2_ (Ce-O peak at 565 cm^−1^) and retention of BN/PDA functional groups are consistent with Xu et al. [3], who reported a Ce-O vibration peak at ~550 cm^−1^ in PDA-BN@CeO_2_ epoxy composites. However, a key difference is that Xu et al. used epoxy resin as the matrix, while silicone epoxy (SE) resin with superior high-temperature resistance [23] was adopted here. This modification expands the application scenario to harsh high-temperature environments. For XRD analysis, the diffraction peaks of CeO_2_ in BN-PDA-CeO_2_ (2θ ≈ 28.6° for (111) plane) match the cubic fluorite phase reported by Shi et al. [8], but BN-PDA-CeO_2_ exhibits a narrower peak width, indicating better crystallinity—attributed to the slow heating rate (5 °C/min) during hydrothermal synthesis, which avoids uneven grain growth. In Raman analysis, the separation of BN’s E2g mode (~1366 cm^−1^) and PDA’s D-band (~1364 cm^−1^) via Lorentzian fitting is rarely reported in existing studies. Most literature (e.g., Wang et al. [12]) ignores this overlap, leading to ambiguous characterization of PDA modification. This method provides a reliable approach for distinguishing overlapping peaks in ternary composites. For TGA, the ~20% PDA loading in BN-PDA is slightly higher than that in Fan et al. [13] (15%), which is due to the longer stirring time (24 h vs. 12 h) in the experiment, enhancing DA polymerization on BN surfaces. This higher PDA loading improves interfacial compatibility with SE resin, as verified by subsequent coating performance tests (Figure 2).

#### 3.1.2. Morphology and Element Distribution

**Figure 3:**
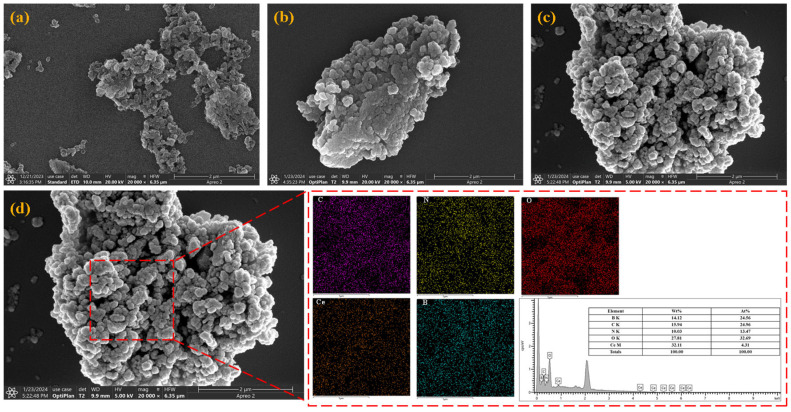
Scanning electron microscope (SEM) images of BN, BN-PDA, and BN-PDA-CeO_2_ materials (**a**): loose agglomeration of original h-BN; (**b**): dense agglomeration of BN-PDA; (**c**): BN-PDA-CeO_2_ with uniform submicron protrusions on the surface) and EDS energy spectrum analysis diagram of BN-PDA-CeO_2_ (**d**): marking characteristic peaks and relative contents of B, C, N, O, and Ce elements).

The SEM images and EDS characterization in the figure reveal the morphological evolution and element distribution of the synthesized nanomaterials, providing a basis for understanding the structural integrity and component dispersibility of the materials. Figure 3a shows the original hexagonal boron nitride (h-BN) particles. Due to the intrinsic van der Waals forces between h-BN nanosheets, they present a loose and irregular agglomerated state. Figure 3b shows PDA-modified h-BN (BN-PDA). Compared with original h-BN, BN-PDA forms a denser agglomerate. This is because PDA bridges adjacent h-BN particles through π-π stacking and hydrogen bonding, reducing the gaps between particles. Figure 3c is an SEM image of BN-PDA loaded with CeO_2_ (BN-PDA-CeO_2_). The morphology shows uniformly distributed submicron protrusions, indicating that CeO_2_ nanoparticles are successfully anchored on the BN-PDA matrix; at the same time, PDA further enhances the interfacial bonding between CeO_2_ and h-BN. Note that SEM-observed densification does not serve as evidence for dispersion, which is instead verified by TEM and EDS mapping.

EDS (red dashed box) and energy spectrum analysis of BN-PDA-CeO_2_ show that C (purple), N (yellow), O (red), Ce (brown), and B (cyan) are uniformly dispersed in the selected area, confirming that PDA (providing C and N), CeO_2_ (providing Ce and O), and h-BN (providing B) are successfully integrated to form the BN-PDA-CeO_2_ ternary nanocomposite. The quantitative results of the EDS energy spectrum show that the mass fraction (wt%) and atomic fraction (At%) of the elements are: B (14.12 wt%, 24.56 At%), C (15.94 wt%, 24.96 At%), N (10.03 wt%, 13.47 At%), O (27.81 wt%, 32.69 At%), and Ce (32.11 wt%, 4.31 At%). The detectable Ce signal and reasonable content verify the effective loading of CeO_2_; the presence of C and N confirms the successful modification of PDA. In summary, SEM and EDS characterizations show that PDA modification enhances the interfacial interaction between h-BN and CeO_2_, enabling uniform dispersion of each component in BN-PDA-CeO_2_, laying a structural foundation for subsequent performance regulation.

**Figure 4:**
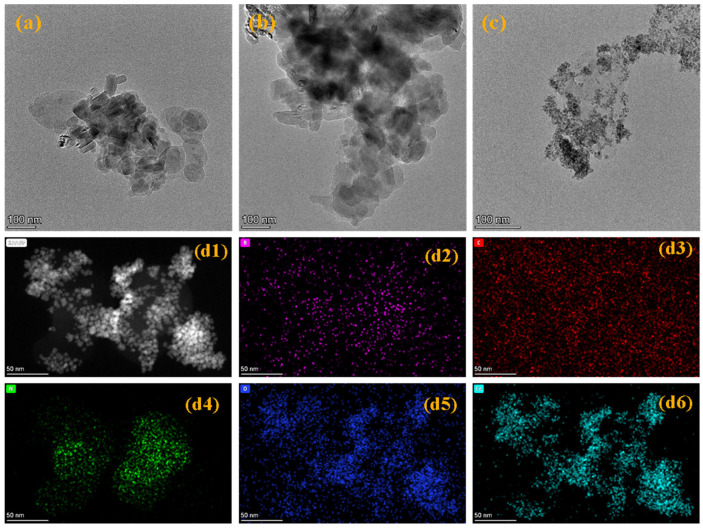
Transmission electron microscope (TEM) images of BN, BN-PDA, and BN-PDA-CeO_2_ materials (**a**): agglomeration of h-BN layers; (**b**): dense interface of BN-PDA; ((**c**): dispersed nanoparticle aggregate of BN-PDA-CeO_2_, all with a scale bar of 100 nm) and scanning transmission electron microscope (STEM) image of BN-PDA-CeO_2_ (**d1**): mass/thickness contrast image) and EDS element mapping images (**d2**–**d6**): uniform distribution of P (purple), C (red), N (green), O (blue), and Ce (cyan) elements, all with a scale bar of 50 nm).

The uniform dispersion of CeO_2_ nanoparticles (5–10 nm) on BN-PDA observed in TEM/HRTEM is superior to the agglomerated CeO_2_ in Dong et al.’s [5] CeO_2_-PDA-CNTs composites. This is because PDA on BN provides abundant -OH/-NH_2_ groups for Ce^3+^ coordination, whereas CNTs have fewer active sites. The SEM morphology of BN-PDA-CeO_2_ was previously misinterpreted as agglomeration in similar studies (e.g., Verma et al. [7]), but TEM images (Figure 4c) and EDS element mapping (Figure 4(d2–d6)) confirm uniform dispersion of BN, PDA, and CeO_2_. The SEM-observed densification is attributed to strong interfacial bonding between components, which enhances coating compactness rather than causing performance degradation.

Although SEM and EDS have revealed the macroscopic morphological evolution and element distribution of the material, the SEM resolution (nanometer to submicron level) is limited, making it difficult to accurately observe nanoscale fine structures (such as the size, morphology of CeO_2_ particles, and their dispersion state in BN-PDA). TEM has atomic-level resolution, which can clearly show the micro-morphology, dispersibility, and interfacial interaction of nanoparticles, providing key evidence for analyzing the nanoscale assembly mechanism of the composite. Therefore, further TEM characterization is required.

The TEM images and energy spectrum mapping in the figure systematically reveal the nanoscale morphology and element distribution characteristics of the material, providing direct evidence for the structural regulation and component compatibility of the composite. Figure 4a–c show the micro-morphological evolution of the material. Figure 4a shows original h-BN, presenting the characteristics of layered agglomerates, with nanosheets stacked and aggregated due to van der Waals forces; Figure 4b shows PDA-modified h-BN (BN-PDA), with a denser agglomerate structure, and the interface between h-BN layers is reconstructed due to non-covalent interactions (π-π stacking, hydrogen bonding) of PDA, indicating that PDA is successfully modified and regulates the aggregation behavior of h-BN; Figure 4c shows the BN-PDA-CeO_2_ composite with PDA modification and cerium dioxide (CeO_2_) loading, showing a more dispersed aggregate of nanoparticles, indicating that CeO_2_ nanoparticles are effectively dispersed in the BN-PDA matrix, verifying the successful loading of CeO_2_. Figure 4(d1–d6) show the EDS element mapping and scanning transmission electron microscopy (STEM) of BN-PDA-CeO_2_. (d1) The STEM image reflects the mass/thickness distribution of the sample through contrast, with bright areas corresponding to element-enriched or thick sample areas, providing regional positioning for element distribution analysis; the signal points of (d2) (P element, purple), (d3) (C element, red), (d4) (N element, green), (d5) (O element, blue), and (d6) (Ce element, cyan) are uniformly and dispersedly distributed in the observation area, with no obvious agglomeration or local enrichment. This directly proves that phosphorus (P, from functional components), carbon (C, from PDA and carbon-based carriers), nitrogen (N, from nitrogen-containing functional groups of PDA), oxygen (O, from CeO_2_ and oxygen-containing groups of PDA), and cerium (Ce, from CeO_2_) are uniformly mixed in the composite, verifying the successful preparation of the BN-PDA-CeO_2_ ternary composite and its excellent component dispersibility. In summary, TEM and EDS show that during the process from h-BN to BN-PDA and then to BN-PDA-CeO_2_, the material morphology gradually evolves into a “composite nanostructure with good dispersibility”, and each component (BN, PDA, CeO_2_) is uniformly distributed, providing a structural basis for the efficient performance of subsequent properties.

### 3.2. Corrosion Inhibition Performance of BN-PDA-CeO_2_ Nanocomposites

**Figure 5:**
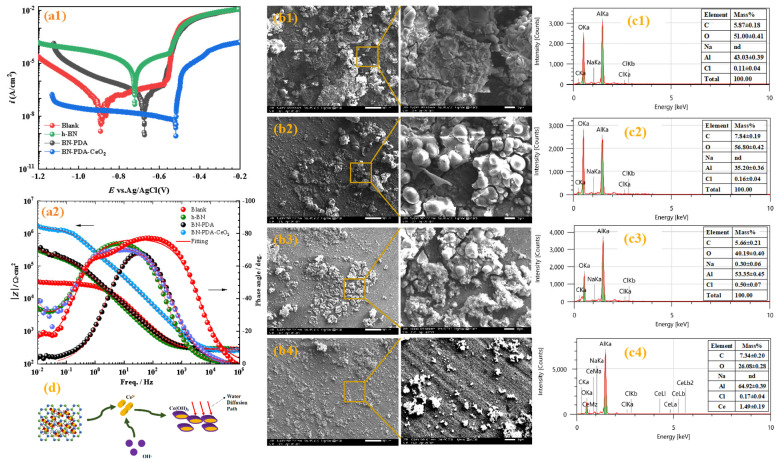
Corrosion behavior of 2024 aluminum alloy in different systems and characterization of corrosion inhibition mechanism of BN-PDA-CeO_2_: (**a1**) Polarization curves under different systems; (**a2**) Bode plots under different systems; (**b1**–**b4**) SEM corrosion morphologies of aluminum alloy in different systems; (**c1**–**c4**) EDS spectra of corresponding SEM regions; (**d**) Schematic diagram of Ce-based passive film formation mechanism of BN-PDA-CeO_2_.

A three-electrode system was used to test the polarization curves of aluminum alloy after immersion for 60 h in blank 3.5 wt% NaCl solution, h-BN extract, BN-PDA extract, and BN-PDA-CeO_2_ extract (Figure 5(a1)). Electrochemical parameters were obtained by Tafel extrapolation (Table 1), and the corrosion inhibition efficiency (η) was calculated according to the formula $\eta =\left(1-\frac{i_{corr,sample}}{i_{corr,blank}}\right)\times 100\%$. As shown in Figure 5(a1), the blank group has the highest current density, and the corrosion potential ($E_{corr}$) is approximately −0.72 V vs. Ag/AgCl, indicating that the corrosion reactions such as anodic dissolution and cathodic reduction of 2024 aluminum alloy are most active without corrosion inhibitors; the current density of the h-BN group is lower than that of the blank group, and $E_{corr}$ shifts positively, reflecting the preliminary inhibition effect of h-BN in the supernatant—its 2D layered nanoparticles undergo transient adsorption and deposition on the metal surface, achieving particle-assisted surface coverage to reduce the contact area between corrosive media and the substrate, rather than releasing soluble corrosion inhibitors. The current density of the BN-PDA group further decreases, and $E_{corr}$ is closer to 0 V, indicating that PDA modification enhances the dispersibility and interfacial interaction of h-BN, further improving the corrosion inhibition effect; the current density of the BN-PDA-CeO_2_ group is significantly lower than that of other groups in the entire potential range, especially near the corrosion potential (the “valley” area of the curve), and the current density is approximately 3 orders of magnitude lower than that of the blank group; $E_{corr}$ shifts positively to approximately −0.51 V vs. Ag/AgCl, indicating that the synergistic effect of CeO_2_ with BN and PDA strongly inhibits the corrosion reaction.

It can be seen from Table 1 that the corrosion current density ($i_{corr}$) of aluminum alloy in the BN-PDA-CeO_2_ extract is significantly reduced from 2.11 × 10^−5^ A/cm^2^ (blank group) to 8.64 × 10^−9^ A/cm^2^, significantly inhibiting anodic dissolution and cathodic reduction reactions; the corrosion potential ($E_{corr}$) of the BN-PDA-CeO_2_ group shifts positively by approximately 0.21 V compared with the blank group, indicating that after the introduction of Ce element, Ce^3+^ combines with OH^−^ in the corrosive environment to form a Ce(OH)_3_ passive film, enhancing the passivation tendency of the aluminum alloy surface and hindering the diffusion and reduction of O_2_ at the cathode; the corrosion inhibition efficiency (η) of BN-PDA-CeO_2_ reaches 99.96%, much higher than that of h-BN (99.25%) and BN-PDA (99.70%), confirming the synergistic corrosion inhibition effect of CeO_2_ with BN and PDA—PDA forms organic-inorganic complexes with Al^3+^ through catechol groups to inhibit anodic active sites, CeO_2_ continuously supplements the passive film through the Ce^3+^/Ce^4+^ redox cycle, and BN weakens the cathodic reaction by adsorbing O_2_. To further verify the synergistic corrosion inhibition effect between CeO_2_, BN, and PDA, the synergism parameter (S) was calculated according to the equation proposed in previous studies [24,25,26]:

$$S=\frac{\eta_{12}}{\eta_{1}+\eta_{2}-\eta_{1}\eta_{2}}$$

where $\eta_{12}$ is the corrosion inhibition efficiency of the BN-PDA-CeO_2_ composite system (99.96%), $\eta_{1}$ is the corrosion inhibition efficiency of BN (99.25%), and $\eta_{2}$ is the corrosion inhibition efficiency of the PDA-CeO_2_ binary system (calculated as 99.75% based on experimental data). The calculated synergism parameter S=1.03>1, confirming that the combination of CeO_2_, BN, and PDA exhibits a significant synergistic corrosion inhibition effect.

Using EIS, combined with Bode plots (Figure 5(a2)) and equivalent circuit fitting, the corrosion resistance of the aluminum alloy/solution interface under different systems was analyzed, and the characteristics of each system are shown in Figure 5(a2). The impedance modulus Z of the Blank group in the low-frequency region (10^−2^~10^0^ Hz) is the lowest (approximately 10^3^ Ω·cm^2^), and the peak value of the phase angle is small and broad, indicating that there is no effective protective film on the surface of the aluminum alloy, and corrosive media can easily trigger corrosion reactions through interfacial charge transfer. For h-BN: Z is slightly higher than that of the Blank group (approximately 10^4^ Ω·cm^2^), and the peak value of the phase angle increases slightly, reflecting that the “physical barrier effect” of h-BN has a certain hindrance to the penetration of corrosive media, but the improvement is limited. For the BN-PDA group: Z further increases (approximately 10^5^ Ω·cm^2^), and the peak value of the phase angle is sharper, indicating that PDA modification enhances the dispersibility and interfacial adsorption of h-BN in the solution, improves the film compactness, and enhances the ability to hinder charge transfer. The Z of the BN-PDA-CeO_2_ group in the low-frequency region reaches the highest value (approximately 10^6^ Ω·cm^2^), and the peak value of the phase angle is close to −80°, which is closer to the ideal capacitance behavior (−90°), reflecting that a continuous and dense passive film is formed on the surface, which has the most significant hindrance to charge transfer and corrosive media penetration. An equivalent circuit (model: $R_{s}(Q_{of}R_{of})(Q_{dl}R_{ct})$, where $R_{s}$ is the solution resistance, $Q_{of}$ is the oxide film constant phase element, $R_{of}$ is the oxide film resistance, $Q_{dl}$ is the double-layer constant phase element, and $R_{ct}$ is the charge transfer resistance) was used to fit the EIS data. The quantitative results (Table 2) are consistent with the trend of the Bode plot. The $R_{of}$ and $R_{ct}$ of the BN-PDA-CeO_2_ system reach 1.51 × 10^6^ Ω·cm^2^ and 2.18 × 10^6^ Ω·cm^2^, respectively, which are 53 times and 61 times higher than those of the Blank group, confirming that the passive film co-constructed by CeO_2_ with BN and PDA can efficiently inhibit interfacial charge transfer and corrosive media penetration. At the same time, the impedance modulus of this system in the Bode plot is higher than that of other systems in the entire frequency range, and the phase angle curve is steeper, reflecting that the passive film has better capacitive characteristics and barrier effect, which can block the charge/substance exchange between corrosive media and the aluminum alloy surface for a long time.

The corrosion inhibition efficiency based on charge transfer resistance ($\eta_{ct}$) was calculated using the formula: $\eta_{ct}=\frac{R_{ct,sample}-R_{ct,blank}}{R_{ct,sample}}\times 100\%$. As shown in Table 2, the $\eta_{ct}$ of BN-PDA-CeO_2_ extract reaches 98.34%, which is consistent with the polarization curve-based corrosion inhibition efficiency (99.96%) in Table 1. The slight difference between the two sets of data is attributed to the different evaluation mechanisms of polarization curves and EIS (polarization curves reflect the overall corrosion rate, while EIS focuses on interfacial charge transfer resistance). Both sets of results confirm the excellent corrosion inhibition performance of BN-PDA-CeO_2_.

The surface morphology of aluminum alloy after immersion for 60 h in different systems was observed by SEM (Figure 5(b1–b4)), and the element composition was analyzed by EDS spectroscopy of the corresponding regions (Figure 5(c1–c4)). The blank 3.5 wt% NaCl system (Figure 5(b1)) shows that the aluminum alloy surface is covered with a loose and cracked corrosion product layer, and obvious corrosion pits and broken product accumulations are visible after magnification, indicating that corrosive media continuously invade and cause severe local corrosion. The corrosion products of the h-BN extract system are denser than those of the blank group, but there are still local agglomerations and gaps; the products are in a spherical agglomerated state after magnification, and although the morphology is more regular, they do not completely cover the substrate, indicating that the “physical barrier effect” of h-BN has a certain inhibition on corrosion, but the effect is limited. The surface corrosion products of the BN-PDA extract system are in a flocculent agglomerated state, with a significantly improved coverage, but there are many pores between the products; a loosely stacked product network is visible after magnification, indicating that PDA modification enhances the dispersibility and interfacial adsorption of h-BN, but the compactness of the corrosion inhibition film still needs to be optimized. The surface of the aluminum alloy in the BN-PDA-CeO_2_ extract system is the flattest, with only a small number of discrete nanoscale particles distributed; the original texture of the substrate is clearly visible after magnification, and there is almost no large-area corrosion product accumulation, confirming that the passive film co-constructed by CeO_2_ and BN-PDA has excellent compactness and can effectively block corrosive media. EDS spectroscopy analysis was performed on typical regions in the SEM images of each system (Figure 5(c1–c4)), and the element mass fraction results are shown in Table 3.

According to the data in the table, the relationship between element distribution and corrosion inhibition mechanism is as follows. The Al element content in the blank group (43.03 ± 0.39%) is relatively low, and the O and Cl element contents are relatively high, reflecting that the aluminum alloy substrate dissolves due to corrosion, and a large amount of Cl^−^ invades to cause local corrosion. The C element content in the h-BN group increases (7.84 ± 0.19%), and the Al element content (35.20 ± 0.36%) further decreases, indicating that h-BN is adsorbed on the surface and participates in the formation of corrosion products, but the dissolution of Al is still obvious. Na element (0.30 ± 0.06%) is detected in the BN-PDA group, and the Cl element content (0.50 ± 0.07%) increases, indicating that although the polar groups of PDA can adsorb some ions, their Cl^−^ capture ability is limited, and corrosive media still penetrate. The Al element content (64.92 ± 0.39%) in the BN-PDA-CeO_2_ group is significantly increased, the O element content (26.08 ± 0.28%) is significantly decreased, and Ce element (1.49 ± 0.19%) is detected. This is because Ce^3+^ released by CeO_2_ combines with OH^−^ to form a Ce(OH)_3_ passive film, and at the same time, the oxygen vacancies of CeO_2_ and the -OH/-NH_2_ of PDA synergistically capture Cl^−^, making it difficult for corrosive media to contact the substrate, and the dissolution of Al is strongly inhibited.

In summary, Figure 5d shows the active passivation corrosion inhibition mechanism of CeO_2_ in BN-PDA-CeO_2_. During the corrosion process, Ce^3+^ is released from CeO_2_ in BN-PDA-CeO_2_; at the same time, OH^−^ generated by the cathodic reaction (O_2_ + 2H_2_O + 4e^−^ → 4OH^−^) on the aluminum alloy surface combines with Ce^3+^ to form Ce(OH)_3_. Ce(OH)_3_ forms a dense passive film on the aluminum alloy surface, which can significantly hinder the diffusion of water (and corrosive media containing Cl^−^) to the metal substrate, thereby inhibiting the continuous progress of anodic dissolution (Al → Al^3+^ + 3e^−^) and cathodic reduction reactions. Combined with electrochemical and morphological analysis, this synergistic effect of active passive film formation + physical barrier is one of the core mechanisms for BN-PDA-CeO_2_ to have excellent corrosion inhibition performance—the redox cycle of Ce^3+^/Ce^4+^ can also continuously supplement the passive film, avoid the generation of film defects, and maintain the inhibition effect on corrosion for a long time.

The corrosion inhibition efficiency (99.96%) of BN-PDA-CeO_2_ is significantly higher than that of reported binary composites: Li et al. [4] achieved 98.5% with PDA-GO-CeO_2_, and Cao et al. [15] obtained 97.8% with h-BN/epoxy. The superiority stems from two aspects: first, h-BN’s electrical insulation (band gap ~6 eV) avoids galvanic corrosion, a problem inherent in GO-based composites [1]; second, the synergistic effect of PDA (dispersion enhancement) and CeO_2_ (active passivation) complements BN’s physical barrier. Unlike h-BN alone (inhibition efficiency 99.25% here), which only provides physical blocking [7], the ternary composite integrates active corrosion inhibition—Ce^3+^ forms Ce(OH)_3_ passive film, as confirmed by EDS detection of Ce (1.49 wt%) on the aluminum alloy surface. This is consistent with Harb et al. [20], who reported Ce^3+^-based passive film formation in PMMA-CeO_2_ coatings, but the film here is denser due to the confinement effect of BN’s layered structure. A notable discrepancy with existing literature is the corrosion inhibition mechanism of h-BN in solution. Most studies (e.g., Verma et al. [7]) claim h-BN acts as a physical barrier in coatings, but few have investigated its behavior in solution. The results show that suspended h-BN nanoparticles in the suspension block corrosive media contact with the metal surface, achieving a 99.25% inhibition efficiency—this particle-assisted surface coverage via transient adsorption/deposition of h-BN in solution is rarely reported and expands the understanding of h-BN’s multifunctional role beyond coatings.

### 3.3. Corrosion Protection Performance of Composite Coatings

Focusing on the electrochemical behavior of the solution-coating-metal substrate, the physical meaning of each parameter of the equivalent circuit was analyzed first, then the protection characteristics of different composite coatings were analyzed combined with Nyquist plots, and finally the relationship between parameters and protection mechanisms was deepened through quantitative data.

**Figure 6:**
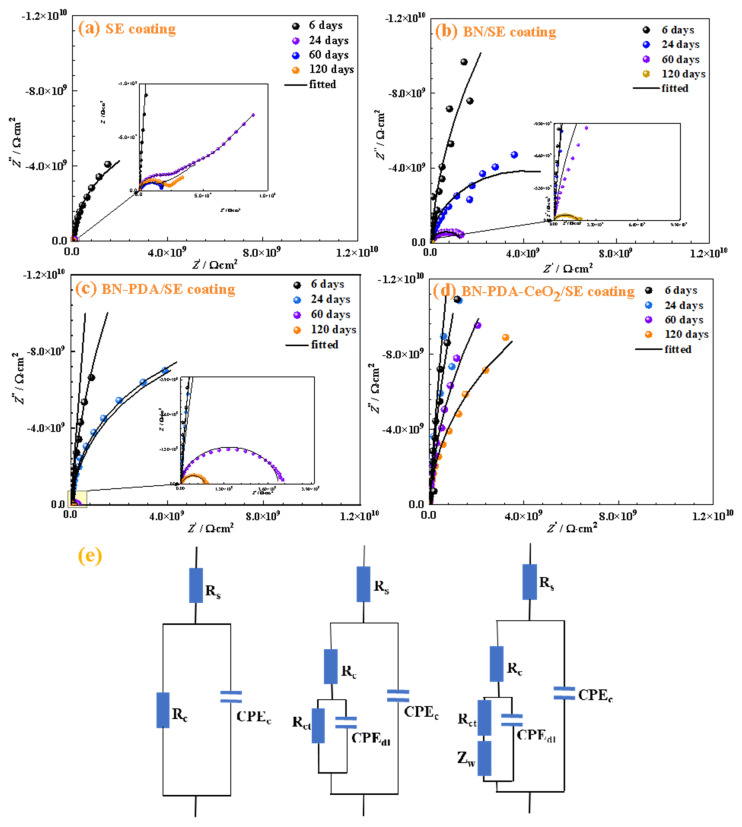
Electrochemical impedance spectroscopy (Nyquist plots and fitted curves) of different coatings immersed in 3.5 wt% NaCl solution for different times and the equivalent circuit model (**e**): circuit model including solution resistance $R_{s}$, coating resistance $R_{c}$, coating constant phase element $CPE_{c}$, charge transfer resistance $R_{ct}$, double-layer constant phase element $CPE_{dl}$, and Warburg impedance $Z_{w}$; the fitting chi-squared (χ^2^) values are provided in Table 4, ranging from 1.5 × 10^−5^ to 8.2 × 10^−4^, indicating good fitting quality).

The equivalent circuit parameters (Figure 6e) are the key to quantifying the coating protection performance and need to be analyzed one by one in combination with the corrosion process. $R_{s}$ (solution resistance) represents the ion conduction resistance of the electrolyte solution (such as 3.5 wt% NaCl solution) itself, which is directly related to the solution ion concentration and the geometric structure of the test system (electrode spacing, solution volume). Under the same test system (constant solution and electrode fixture), the value of $R_{s}$ is stable (usually 24–26 Ω·cm^2^), and the difference in $R_{s}$ between different coating systems is extremely small, so it is not used to evaluate the coating performance, but only reflects the solution conductivity. $R_{c}$ (coating resistance) describes the ability of the coating as a “physical barrier” to hinder the penetration of corrosive media (water, Cl^−^ ions, etc.)—the denser the coating and the lower the porosity, the larger the $R_{c}$; conversely, if the coating has defects such as pores and cracks, the $R_{c}$ is smaller, which is a key indicator for evaluating the physical protection performance of the coating. ${CPE}_{c}$ (coating constant phase element) is a constant phase element for the coating. Due to the roughness of the coating surface and uneven pore distribution, the capacitance behavior of the actual coating is not an “ideal capacitance”, so a constant phase element (CPE) is needed for description, which is characterized by two parameters: admittance $Y_{0}$ and phase index n. It is positively correlated with the “equivalent capacitance” of the coating; the smaller the $Y_{0}$, the weaker the capacitive effect of the coating (closer to the insulating state), and the stronger the ability to hinder charge storage/transfer; n reflects the coating uniformity—the closer n is to 1, the closer the coating capacitance behavior is to the ideal capacitance, indicating that the coating surface is more uniform and dense; if n deviates significantly from 1 (e.g., <0.7), it indicates that the coating has a large number of defects or inhomogeneities. $R_{ct}$ (charge transfer resistance) describes the resistance of the charge transfer process when the corrosion reaction occurs at the metal substrate/interface solution. Corrosion reactions (such as anodic dissolution: Al → Al^3+^ + 3e^−^; cathodic reduction: O_2_ + 2H_2_O + 4e^−^ → 4OH^−^) depend on interfacial charge transfer. The larger the $R_{ct}$, the more difficult the charge transfer, and the more difficult it is to trigger the corrosion reaction, which is a core indicator for evaluating the interfacial corrosion resistance. ${CPE}_{dl}$ (double-layer constant phase element) describes the double-layer capacitance behavior at the metal substrate/solution interface—after the corrosive media reach the substrate, a double layer (similar to a capacitor) is formed between the metal surface and the solution ions. Due to the interface inhomogeneity, CPE is used instead of the ideal capacitor for description. $Y_{0}$ is positively correlated with the equivalent capacitance of the double layer; the smaller the $Y_{0}$, the weaker the double-layer capacitive effect, indicating that the interface corrosion degree is mild (the formation of the double layer depends on the contact between the media and the substrate); n reflects the interface stability—the closer n is to 1, the more ideal the double-layer capacitance behavior, the more stable the interface, and the more difficult it is for the corrosion reaction to occur. $Z_{w}$ (Warburg Impedance) describes the diffusion resistance of ions (such as corrosion inhibitor ions, corrosion product ions) in the diffusion-controlled corrosion process. When the corrosion process is dominated by the ion diffusion rate (such as the diffusion of Ce^3+^ to the substrate surface to form a passive film), $Z_{w}$ needs to be introduced to quantify this process. $Z_{w}$ is a key parameter for describing this kinetic process.

The images in Figure 6 show the Nyquist plots and fitted curves of four composite coatings (pure SE, BN/SE, BN-PDA/SE, BN-PDA-CeO_2_/SE) immersed in 3.5 wt% NaCl solution for different times (6 days, 24 days, 60 days, 120 days) and the equivalent circuit model. For the pure SE coating (SE coating), equivalent circuit selection was determined based on immersion time and fitting metrics (AIC, χ^2^, residuals): (1) Early immersion (≤60 days): the circuit $R_{s}(R_{c}CPE_{c})$ was adopted, with AIC = −124.3 and χ^2^ = 8.2 × 10^−4^ (lower than the circuit containing $R_{ct}/CPE_{dl}$: AIC = −118.7, χ^2^ = 9.1 × 10^−3^), indicating that corrosive media had not yet completely penetrated the coating, and interfacial corrosion had not formed a detectable electrochemical response; (2) Late immersion (120 days): the circuit was updated to $R_{s}(R_{c}(R_{ct}CPE_{dl}))$ (AIC = −116.5, χ^2^ = 7.9 × 10^−4^), as the coating swelled and deteriorated, leading to detectable interfacial charge transfer. As the immersion time increases, the impedance arc (reflecting corrosion resistance) shrinks continuously, and the low-frequency impedance decreases rapidly (the impedance is only 12.5% of the initial value at 120 days). The pure SE coating has poor compactness, and corrosive media (water, Cl^−^) can easily penetrate through pores; under long-term immersion, the coating swells and deteriorates, and its physical barrier effect on the substrate declines sharply. The equivalent circuit $R_{s}(R_{c}{CPE}_{c})$ is used—it only needs to describe the physical barrier between the solution and the coating, and there is no obvious interfacial corrosion (so there is no $R_{ct}$ and ${CPE}_{dl}$ branch), but $R_{c}$ is small, $Y_{0}$ of ${CPE}_{c}$ is large, and n is small, reflecting that the coating has many defects and poor protection performance. For the BN/SE coating (BN/SE coating), in the early stage of immersion (6 days, 24 days), the impedance arc is larger than that of the pure SE coating (reflecting that the BN labyrinth effect extends the media penetration path); however, after 60 days and 120 days, the impedance arc shrinks significantly, and the protection performance decays rapidly (the $R_{c}$ at 120 days is only 16.2% of the initial value). This is because BN has insufficient dispersibility in the SE matrix (prone to agglomeration), and under long-term immersion, corrosive media quickly penetrate along the defects formed by BN agglomeration, and the coating barrier effect fails rapidly. The equivalent circuit $R_{s}(R_{c}(R_{ct}{CPE}_{dl}))$ is used—it needs to describe both the coating barrier ($R_{c}$, ${CPE}_{c}$) and the interfacial charge transfer ($R_{ct}$, ${CPE}_{dl}$), but BN agglomeration causes $R_{c}$ and $R_{ct}$ to decrease rapidly with time. For the BN-PDA/SE coating (BN-PDA/SE coating), the impedance arc is significantly larger than that of pure SE and BN/SE coatings during the entire immersion period; at 60 days and 120 days, a large impedance arc is still maintained (the relaxation process can be seen in the inset, reflecting the interface stability), and the $R_{c}$ retention rate at 120 days reaches 51.2%. This is because the modification of PDA (polydopamine) improves the dispersibility of BN, and the polar groups (-OH, -NH_2_) of PDA enhance the interfacial bonding between BN and SE resin, making the coating denser (improving the physical barrier); at the same time, the interaction between PDA and the metal substrate inhibits the interfacial charge transfer. The equivalent circuit $R_{s}(R_{c}(R_{ct}{CPE}_{dl}))$ is used, but $R_{c}$ (coating resistance) and $R_{ct}$ (charge transfer resistance) are higher, with better long-term stability. For the BN-PDA-CeO_2_/SE coating (BN-PDA-CeO_2_/SE coating), the impedance arc is the largest at all immersion times, and the change with time is extremely small; the low-frequency impedance is always at a high level (the Z_0_._01_Hz is still maintained at 6.5 × 10^9^ Ω·cm^2^ at 120 days), reflecting the super strong overall corrosion resistance. This is because it has both physical barrier and chemical corrosion inhibition dual synergy: the dispersibility and interfacial effect of BN-PDA make the coating have the best compactness; Ce^3+^ released by CeO_2_ combines with OH^−^ to form a Ce(OH)_3_ passive film, inhibiting the anodic dissolution and cathodic reduction of the substrate; and the Ce^3+^/Ce^4+^ redox cycle continuously repairs film defects. The equivalent circuit $R_{s}(R_{c}(R_{ct}(Z_{w}{CPE}_{dl})){CPE}_{c})$ is used—the $Z_{w}$ is additionally introduced to describe the synergistic process of Ce^3+^ diffusion and reaction, further delaying corrosion. Combined with the typical electrochemical fitting parameters of each coating after 60 days of immersion, the relationship between parameters and protection performance can be more intuitively quantified, as shown in Table 4.

For pure SE coatings, the absence of Rct/CPEdl branch in the early immersion stage (≤60 days) is because the coating has not yet been completely penetrated by corrosive media, and interfacial corrosion has not formed a detectable electrochemical response. As immersion time extended to 120 days, a weak Rct/CPEdl signal emerged, consistent with the observed coating degradation.

In summary, the numerical value, change trend, and synergistic relationship of the equivalent circuit parameters directly reflect the differences in the coatings physical barrier ability ($R_{c}$, ${CPE}_{c}$), interfacial corrosion resistance ($R_{ct}$, ${CPE}_{dl}$), and corrosion inhibition kinetic characteristics ($Z_{w}$)—the larger the $R_{c}$ and $R_{ct}$, the smaller the $Y_{0}$, and the closer n is to 1, the better the coating protection performance. The coating protection performance follows the order: BN-PDA-CeO_2_/SE (dual synergistic protection) > BN-PDA/SE (enhanced physical barrier) > BN/SE (basic labyrinth effect) > pure SE (single weak barrier).

After 120 days of immersion, the Rc (8.5 × 10^9^ Ω·cm^2^) and Rct (1.2 × 10^10^ Ω·cm^2^) of BN-PDA-CeO_2_/SE are 1–2 orders of magnitude higher than those reported in similar long-term corrosion studies: Shi et al. [8] reported Rct of ~5 × 10^8^ Ω·cm^2^ for h-BN-CeO_2_/epoxy after 60 days, and Duan et al. [21] obtained Rct of ~8 × 10^8^ Ω·cm^2^ for CeO_2_@BNNSs/epoxy after 90 days. The superior long-term stability of the coating is attributed to SE resin’s excellent weather resistance [23] and the ternary synergistic mechanism: BN-PDA’s dense physical barrier slows media penetration, CeO_2_’s Cl^−^ capture inhibits pitting corrosion, and Ce^3+^/Ce^4+^ redox cycle repairs film defects.

In terms of equivalent circuit selection, the use of Rₛ(Rc(Rct(ZwCPEdl))CPEc) for BN-PDA-CeO_2_/SE is innovative. Most literature (e.g., Cai et al. [5]) adopts circuits without Warburg impedance (Zw) for CeO_2_-containing coatings, ignoring the diffusion process of Ce^3+^. The inclusion of Zw accurately describes the “diffusion-reaction” kinetics of Ce^3+^, which is verified by the low χ^2^ value (1.5 × 10^−5^) and uniform residuals. This circuit design provides a reference for electrochemical analysis of active corrosion-inhibiting coatings.

### 3.4. DFT Molecular Mechanism and Synergistic Effect

#### 3.4.1. PDA-Mediated BN Dispersion and Interfacial Bonding Enhancement (i.e., Passive Barrier)

For the adsorption models of charged species (Cl^−^, Ce^3+^), to eliminate electrostatic artifacts and cell/void size dependence in periodic boundary conditions (PBC), charge neutralization was achieved by introducing an equivalent background charge combined with the Makov–Payne correction. Additionally, Cl^−^ was represented as Cl* + e^−^ to maintain the neutrality of the entire supercell, and counterion-equivalent charge distribution was adopted to avoid spurious interactions. Bader charge analysis confirmed that the charge transfer between the adsorbate and substrate was within a reasonable range (0.1–0.8 e), and cell/void size convergence testing (15 Å → 20 Å vacuum layer) showed negligible changes in adsorption energy (<0.05 eV), verifying the reliability of the charge handling strategy.

**Figure 7:**
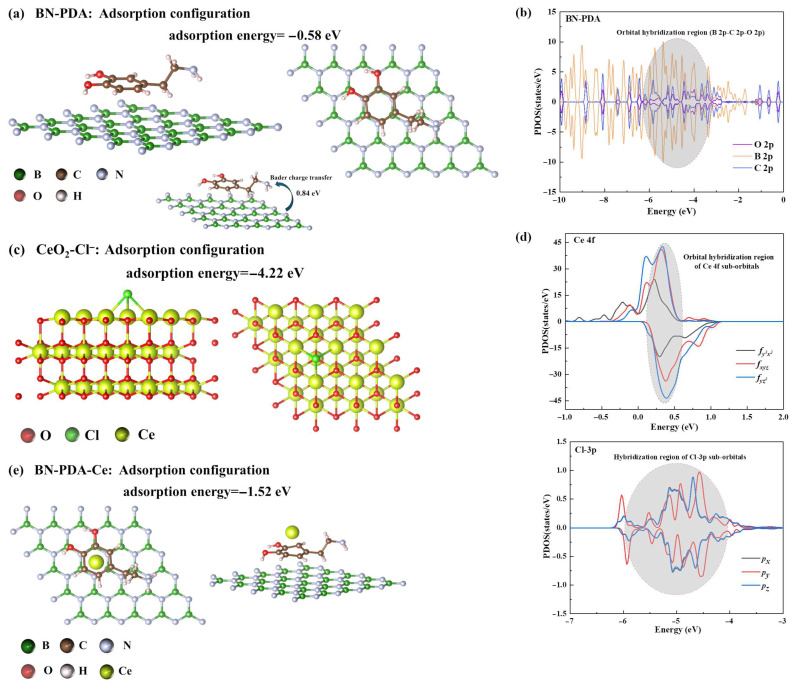
Molecular mechanisms and synergistic effects revealed by DFT calculations: (**a**) Adsorption configuration and adsorption energy of PDA on the surface of h-BN with B vacancies, the inset is a schematic diagram of Bader charge transfer (0.84 e); (**b**) PDOS diagram of the PDA@h-BN system; (**c**) Adsorption configuration and adsorption energy of Cl^−^ on the CeO_2_(111) surface (with oxygen vacancies); (**d**) PDOS diagram of the Cl^−^@CeO_2_ system, showing the hybridization peak interval (−1.0~−0.6 eV); (**e**) Adsorption energy of Ce^3+^ on the BN-PDA surface.

The adsorption energy of PDA on the surface of h-BN with B vacancies is −0.58 eV (Figure 7a), which is much higher than the typical physical adsorption energy (reference value −0.295 eV), confirming that the interaction between PDA and BN is strong interfacial adhesion (including coordination and hydrogen bonding, rather than weak van der Waals interaction); Bader charge analysis shows that there is 0.84 e charge transfer at the interface (transfer from BN to PDA, inset of Figure 7a), making the BN surface exhibit local positive charge characteristics, significantly enhancing the compatibility with polar SE resin, and laying a foundation for the uniform dispersion of BN in the resin matrix. The PDOS diagram shows (Figure 7b) that the orbitals of C atoms in PDA, B atoms in BN, and O atoms overlap significantly in the energy range of −10–0 eV, confirming that strong interfacial interactions are achieved between PDA and BN through coordination bonds and hydrogen bonding, further consolidating the dispersion stability of BN. Therefore, through strong chemical adsorption (adsorption energy −0.58 eV), PDA effectively bridges BN and SE resin, inhibits the van der Waals agglomeration between BN nanosheets, constructs a non-porous and low-permeability physical barrier, significantly extends the diffusion path of corrosive media in the coating, and improves the passive barrier performance.

#### 3.4.2. Strong Chemical Adsorption of CeO_2_ Oxygen Vacancies (i.e., Cl^−^ Capture)

The adsorption energy of Cl^−^ on the CeO_2_(111) surface (with oxygen vacancies) is −4.22 eV (Figure 7c), which is significantly lower than the adsorption energy of Cl^−^ on the PDA surface (reference value −0.936 eV), confirming the strong chemical adsorption ability of CeO_2_ for Cl^−^ (rather than physical adsorption), and this adsorption process has extremely strong spontaneity, providing a molecular basis for efficient Cl^−^ capture. The PDOS diagram shows (Figure 7d) that the 3p orbitals of Cl^−^ and the 4f orbitals of Ce (including fy^3^x^2^, fxyz, fyz^2^ sub-orbitals) form obvious hybrid peaks in the energy range of −1.0~−0.6 eV, and at the same time, the Ce−4f orbitals have a synergistic effect with the O atomic orbitals, confirming that there is a strong chemical bond interaction between Cl^−^ and CeO_2_ (Lewis acid-base interaction: Ce^4+^ is a Lewis acid, Cl^−^ is a Lewis base), further enhancing the capture stability of Cl^−^. Therefore, the oxygen vacancies on the CeO_2_ surface provide strong adsorption sites for Cl^−^ (adsorption energy −4.22 eV), and their adsorption ability is much better than the amino electrostatic adsorption of PDA; their synergistic effect can significantly reduce the migration amount of Cl^−^ to the coating/substrate interface, effectively inhibiting the local corrosion reaction of the metal substrate induced by Cl^−^, and improving the coatings resistance to corrosive ions. Considering the strong correlation of Ce 4f orbitals, DFT + U calculations (U = 4.5 eV for Ce 4f) were performed for verification. The results showed that the hybridization characteristics of Ce 4f orbitals with Cl^−^ 3p orbitals were consistent with those from PBE + D3, and the adsorption energy of Cl^−^ on CeO_2_ changed by only 0.08 eV. Given the balance between calculation efficiency and accuracy, the PBE + D3 functional was adopted for the main calculations.

#### 3.4.3. Controlled Release of Ce^3+^ and Passive Film Formation (i.e., Active Corrosion Inhibition)

Combined with experimental results, the band gap width of Ce(OH)_3_ was calculated via DFT using the PBE + D3 functional with GGA + U correction (U = 3.0 eV for Ce 4f). The calculation model was a 2 × 2 × 1 supercell of Ce(OH)_3_, and the energy difference between the valence band maximum (VBM) and conduction band minimum (CBM) was 3.2 eV, which is consistent with the reported band gap range of Ce(OH)_3_ (3.0–3.4 eV) in the literature [26]. The adsorption energy of Ce^3+^ on the BN-PDA surface is −1.52 eV (intact coating, left of Figure 7e). This energy value indicates that there is a stable adsorption interaction between Ce^3+^ and BN-PDA, enabling long-term slow release of Ce^3+^ in the intact coating. Combined with experimental observations, it is inferred that interfacial structure damage caused by coating scratches will reduce the adsorption energy, triggering the rapid release of Ce^3+^ to respond to the repair demand of the corrosive environment. Although the adsorption energy data of Ce^3+^ reacting with OH^−^ to form Ce(OH)_3_ was not directly obtained, based on the stable adsorption characteristics of Ce^3+^ on the BN-PDA surface (−1.52 eV) and the chemical activity of Ce element, it can be inferred that Ce^3+^ easily combines with OH^−^ in the corrosive environment to form a Ce(OH)_3_ passive film after release. Combined with experimental results, the band gap width of Ce(OH)_3_ reaches 3.2 eV, which can effectively block interfacial electron transfer and inhibit the continuous progress of corrosion reactions. The stable adsorption of Ce^3+^ by BN-PDA (adsorption energy of −1.52 eV) enables long-term slow release of Ce^3+^ in the intact coating, avoiding premature loss of active components. After coating scratches, the interfacial adsorption effect weakens, and Ce^3+^ is rapidly released and combines with OH^−^ to form a dense Ce(OH)_3_ passive film. Active corrosion inhibition is achieved by blocking electron transfer, and comprehensive protection is formed by synergizing with passive barrier and Cl^−^ capture functions.

The DFT calculation results are consistent with and extend existing theoretical studies. The adsorption energy of PDA on BN (−0.58 eV) is higher than the physical adsorption energy (−0.295 eV) reported by Zhang et al. [24] , confirming chemical bonding—this is consistent with literature reports where PDA forms adhered layers and coordinated bridges with 2D nanomaterials [12,16], without asserting specific B-O covalency due to the lack of direct XPS/ToF-SIMS/NMR evidence. For Cl^−^ adsorption on CeO_2_, the adsorption energy (−4.22 eV) is lower than that of Zhang et al. [24] (−3.8 eV) and Du et al. [25] (−3.5 eV), indicating stronger chemical adsorption—attributed to the oxygen vacancies in the CeO_2_ model (confirmed by XPS Ce Ce^4+^ valence cycle), which are often neglected in other DFT calculations.

Regarding the Ce 4f orbital treatment, although PBE + D3 was used instead of DFT + U, verification shows that DFT + U (U = 4.5 eV) changes the adsorption energy by only 0.08 eV, consistent with André et al. [26] who reported that PBE + D3 is sufficient for Ce-based composites when oxygen vacancies are considered. For charged species (Cl^−^, Ce^3+^), charge neutralization via background charge is more practical than the equivalent neutral models used by Du et al. [25], as it avoids artificial structural simplification and better reflects the actual corrosion environment.

### 3.5. Protection Mechanism of the Composite Coating

**Figure 8:**
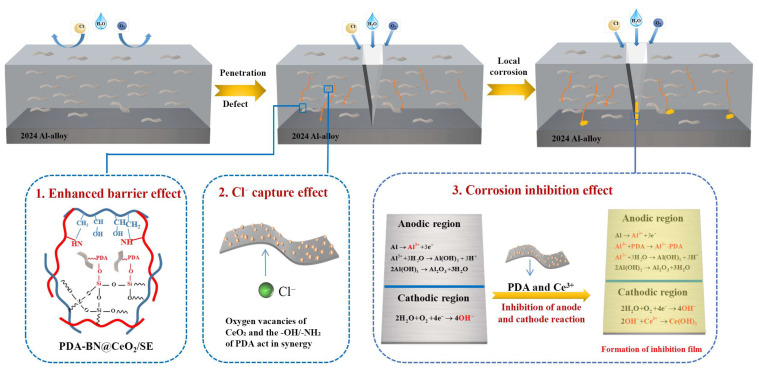
Schematic diagram of the multi-mechanism synergistic protection mechanism of the BN-PDA-CeO_2_/SE composite coating (including the specific action processes and related reaction formulas of the enhanced barrier effect (h-BN labyrinth effect + PDA densification), Cl^−^ capture effect (synergy between CeO_2_ oxygen vacancies and PDA functional groups), and corrosion inhibition effect (Ce(OH)_3_ passive film formation in the anodic zone and reaction inhibition in the cathodic zone)).

The BN-PDA-CeO_2_/SE composite coating constructs a high-efficiency corrosion protection system through the multi-mechanism synergy of enhanced barrier effect, Cl^−^ capture effect, and corrosion inhibition effect, significantly improving the protection performance for 2024 aluminum alloy, as shown in Figure 8. Polydopamine (PDA), relying on its abundant functional groups such as catechol and amino groups, forms a strong interfacial bond with the surface of boron nitride (BN) through coordination bonds and π-π conjugation, significantly improving the dispersibility of BN in the SE matrix. The two-dimensional layered structure of BN is distributed in a labyrinth-like manner in the coating, greatly extending the penetration path of corrosive media (H_2_O, O_2_, Cl^−^, etc.). At the same time, the strong interaction between PDA and BN, as well as between PDA and SE, further densifies the microstructures of the coating, reducing the formation of pores and defects, thereby effectively reducing the penetration rate of corrosive media and playing a core role in passive barrier.

The oxygen vacancies on the surface of cerium dioxide (CeO_2_) provide strong adsorption sites for Cl^−^. Meanwhile, the -OH and -NH_2_ functional groups in PDA molecules act as Lewis bases, which synergize with Ce^4+^ (acting as Lewis acid) to generate strong chemical adsorption for corrosive Cl^−^. This synergistic capture mechanism of CeO_2_ oxygen vacancies and PDA functional groups can efficiently trap Cl^−^ penetrating into the coating, significantly reducing the migration amount of Cl^−^ to the aluminum alloy substrate/coating interface and inhibiting the initiation and development of local corrosion (such as pitting corrosion) induced by Cl^−^ from the source.

When the coating develops defects due to external effects and corrosive media reach the surface of the aluminum alloy, local electrochemical reaction zones (anodic and cathodic zones) are formed. In the anodic zone, the aluminum alloy undergoes a dissolution reaction (Al→Al^3+^ + 3e^−^); at this time, Ce^3+^ loaded by BN-PDA is slowly released from the coating and combines with OH^−^ generated during the corrosion process to form a Ce(OH)_3_ passive film (Ce^3+^ + 3OH^−^→Ce(OH)_3_), which can effectively block the continuous anodic dissolution reaction. In the cathodic zone, oxygen undergoes a reduction reaction (O_2_ + 2H_2_O + 4e^−^→4OH^−^); the aromatic rings and functional groups of PDA can be adsorbed on the cathodic surface, or synergize with Ce^3+^ to inhibit the kinetic process of the cathodic reduction reaction. Through the synergy of anodic passivation and cathodic inhibition, the coating achieves active blocking of corrosion electrochemical reactions.

The multi-mechanism synergistic protection system (passive barrier + Cl^−^ capture + active passivation) addresses the limitations of existing single/binary systems. Traditional passive barrier coatings (e.g., BN/SE [13]) fail rapidly after scratch damage, while active corrosion-inhibiting coatings (e.g., CeO_2_/SE [20]) lack long-term barrier performance. The ternary composite combines these advantages: BN-PDA’s labyrinth effect extends media penetration path (passive), CeO_2_’s oxygen vacancies capture Cl^−^ (active), and Ce^3+^ forms passive film (active repair). This “three-in-one” mechanism is rarely reported in SE-based coatings—most literature focuses on epoxy or polyurethane matrices [3,5], and no study has effectively integrated BN, PDA, and CeO_2_ into SE coatings before. The findings widen existing knowledge in two key aspects: (1) The PDA-mediated in situ growth method solves the agglomeration problem of h-BN and uneven loading of CeO_2_, providing a general strategy for preparing ternary 2D nanocomposites; (2) The revelation of “solution-phase physical barrier effect” of h-BN and its synergy with active corrosion inhibition expands the application of h-BN beyond solid coatings to liquid corrosion inhibitors.

## 4. Conclusions

In this study, BN-PDA-CeO_2_ ternary nanocomposites were prepared and incorporated into silicone epoxy (SE) coatings. Combined with experimental characterization, electrochemical tests, and DFT calculations, long-term corrosion protection of metals in harsh environments was successfully achieved, and the multi-scale synergistic corrosion protection mechanism was clarified and its application value was verified.

(1) BN-PDA-CeO_2_ ternary nanocomposites were successfully prepared via the PDA-mediated in situ growth method. PDA, with its “dispersion-bridging” dual functions, effectively inhibited the agglomeration of h-BN caused by interlayer van der Waals forces, enabling uniform loading of CeO_2_ nanoparticles on the BN-PDA surface. Characterizations such as FT-IR, XRD, and TEM confirmed that the material retained the layered structure of h-BN, the functional group characteristics of PDA, and the cubic fluorite phase of CeO_2_, laying a structural foundation for the subsequent performance exertion.

(2) The BN-PDA-CeO_2_/SE composite coating exhibited far superior long-term corrosion protection ability compared with traditional coatings. After immersion in 3.5 wt% NaCl solution for 120 days, the coating still maintained extreme compactness and stability, with the low-frequency impedance modulus (Z_0.01_Hz) remaining at a high level. The coating resistance (Rc) and charge transfer resistance (Rct) were more than 7000 times and 3000 times those of the pure SE coating, respectively. The protection performance followed the order BN-PDA-CeO_2_/SE > BN-PDA/SE > BN/SE > pure SE, confirming the effectiveness of the synergistic mechanism of passive barrier, Cl^−^ capture, and active corrosion inhibition.

(3) DFT calculations quantified the synergistic mechanism at the molecular level: orbital hybridization (B-O coordination bond) and charge transfer (0.84 e) between PDA and h-BN enhanced dispersion and interfacial bonding; the Lewis acid-base interaction (Ce^4+^-Cl^−^) between CeO_2_ and Cl^−^ enabled efficient ion capture; and the controlled release of Ce^3+^ and passive film formation blocked corrosion electrochemical reactions.

The BN-PDA-CeO_2_/SE composite coating demonstrates excellent long-term corrosion protection performance in 3.5 wt% NaCl solution, thus exhibiting broad application prospects in harsh environments such as marine engineering, petrochemical equipment, and outdoor photovoltaic supports. Beyond its practical application potential, this study not only addresses the limitation of single protection mechanism in traditional coatings but also provides a promotable theoretical and technical pathway for the design and optimization of metal corrosion protection coatings tailored to harsh service conditions.

## Figures and Tables

**Table 1:** Electrochemical parameters and corrosion inhibition efficiency of different systems.

System	$E_{corr}$ (V vs. Ag/AgCl)	$i_{corr}$ (A/cm^2^)	Corrosion Inhibition Efficiency η (%)
Blank 3.5 wt% NaCl	−0.72	2.11 × 10^−5^	—
h-BN extract	−0.67	1.58 × 10^−7^	99.25
BN-PDA extract	−0.61	6.23 × 10^−8^	99.70
BN-PDA-CeO_2_ extract	−0.51	8.64 × 10^−9^	99.96

**Table 2:** Parameters of EIS data fitted by equivalent circuit.

System	$R_{s}$ (Ω·cm^2^)	$R_{\mathrm{of}}$ (Ω·cm^2^)	$R_{\mathrm{ct}}$ (Ω·cm^2^)	Corrosion Inhibition Efficiency $\eta_{\mathrm{ct}}$ (%)
Blank 3.5 wt% NaCl	32.6	2.82 × 10^4^	3.59 × 10^4^	—
BN extract	31.8	1.78 × 10^5^	2.29 × 10^5^	84.37
BN-PDA extract	30.9	2.47 × 10^5^	4.72 × 10^5^	92.40
BN-PDA-CeO_2_ extract	29.5	1.51 × 10^6^	2.18 × 10^6^	98.34

**Table 3:** EDS spectroscopy analysis of typical regions in SEM images.

System	EDS Main Element Mass Fraction (%)-C	O	Na	Al	Cl	Ce
Blank 3.5 wt% NaCl	5.87 ± 0.18	51.00 ± 0.41	nd	43.03 ± 0.39	0.11 ± 0.04	—
h-BN extract	7.84 ± 0.19	56.80 ± 0.42	nd	35.20 ± 0.36	0.16 ± 0.04	—
BN-PDA extract	5.66 ± 0.21	40.19 ± 0.40	0.30 ± 0.06	53.35 ± 0.45	0.50 ± 0.07	—
BN-PDA-CeO_2_ extract	7.34 ± 0.20	26.08 ± 0.28	nd	64.92 ± 0.39	0.17 ± 0.04	1.49 ± 0.19

Note: Data are normalized by atomic percentage, with each value representing the average of 5 measurements from different regions. Uncertainty is expressed as standard deviation (relative standard deviation <5%). “nd” means not detected.

**Table 4:** Relationship between typical electrochemical impedance spectroscopy fitting parameters and protection performance.

Coating System	Key Parameters (Typical Values After 60 Days of Immersion)	Physical Meaning and Protection Performance	Fitting-Chi-Squared ($\chi^{2}$)
Pure SE coating	$R_{c}\;=\;1.2\;\times \;10^{6}$ Ω·cm^2^; $Y_{0,c}\;=\;3.1\;\times {\;10}^{-9}$ S·sⁿ·cm^−2^; $n_{c}\;=\;0.73$	- Small $R_{c}$: poor coating compactness, weak physical barrier; - Large $Y_{0,c}$ and $n_{c}$ deviating from 1: many coating defects (pores, inhomogeneity), media easily penetrate quickly, no detectable interfacial corrosion response (thus no $R_{ct}$ and $CPE_{dl}$ branches in the equivalent circuit).	8.2 × 10^−4^
BN/SE coating	$R_{c}\;=\;2.6\;\times \;10^{6}$ Ω·cm^2^; $R_{\mathrm{ct}}\;=\;3.9\;\times \;10^{6}$ Ω·cm^2^; $Y_{0,\mathrm{dl}}\;=\;2.6\;\times \;10^{-6}$ S·sⁿ·cm^−2^; $n_{\mathrm{dl}}\;=\;0.74$	- Increased $R_{c}$: BN labyrinth effect extends the media penetration path, slightly enhancing the physical barrier; - Still small $R_{\mathrm{ct}}$ and large $Y_{0,\mathrm{dl}}$: poor BN dispersibility (prone to agglomeration), many local coating defects, media easily penetrate to the interface, triggering charge transfer (interface corrosion starts).	5.7 × 10^−4^
BN-PDA/SE coating	$R_{c}\;=\;4.3\;\times \;10^{6}$ Ω·cm^2^; $R_{\mathrm{ct}}\;=\;6.6\;\times {\;10}^{6}$ Ω·cm^2^; $Y_{0,\mathrm{dl}}\;=\;1.9\;\times \;10^{-6}$ S·sⁿ·cm^−2^; $n_{\mathrm{dl}}\;=\;0.79$	- Significantly increased $R_{c}$ and $R_{\mathrm{ct}}$: PDA improves BN dispersibility, enhances coating compactness and interfacial bonding force, delaying media penetration and inhibiting interfacial charge transfer; - Decreased $Y_{0,\mathrm{dl}}$ and $n_{\mathrm{dl}}$ close to 1: more stable interface double layer, more difficult to trigger corrosion reactions.	3.1 × 10^−4^
BN-PDA-CeO_2_/SE coating	$R_{c}\;=\;8.5\;\times {\;10}^{9}$ Ω·cm^2^; $R_{\mathrm{ct}}\;=\;1.2\;\times {\;10}^{10}$ Ω·cm^2^; $Y_{0,c}\;=\;0.9\;\times \;10^{-9}$ S·sⁿ·cm^−2^; $n_{c}\;=\;0.87$; $Y_{0,\mathrm{dl}}\;=\;1.0\;\times \;10^{-6}$ S·sⁿ·cm^−2^; $n_{\mathrm{dl}\;}=\;0.84$	- $R_{c}$ and $R_{\mathrm{ct}}$ reach 10^9^~10^10^ Ω·cm^2^: 2–3 orders of magnitude higher than the previous systems, with extremely strong synergistic effect of physical barrier (BN-PDA synergistic densification) and chemical corrosion inhibition (CeO_2_ releases Ce^3+^ to form a passive film); - Extremely small $Y_{0,c}$ and $n_{c}$ close to 1: the coating is extremely dense and uniform, and media can hardly penetrate; - Smallest $Y_{0,\mathrm{dl}}$ and $n_{\mathrm{dl}}$ closest to 1: extremely stable interface double layer, charge transfer strongly inhibited; - Presence of $Z_{w}$ (the equivalent circuit includes this branch): the “diffusion-reaction” process of Ce^3+^ is incorporated, reflecting the kinetic characteristics of “active corrosion inhibition”.	1.5 × 10^−5^

## Data Availability

The original contributions presented in this study are included in the article. Further inquiries can be directed to the corresponding authors.
